# Butyrate, a metabolite of intestinal bacteria, enhances sleep

**DOI:** 10.1038/s41598-019-43502-1

**Published:** 2019-05-07

**Authors:** Éva Szentirmai, Nicklaus S. Millican, Ashley R. Massie, Levente Kapás

**Affiliations:** 1 Elson S. Floyd College of Medicine, Department of Biomedical Sciences, Washington State University, Spokane Washington, United States of America; 2 Sleep and Performance Research Center, Washington State University, Spokane Washington, United States of America

**Keywords:** Sleep, Circadian regulation

## Abstract

Emerging evidence suggests that the intestinal microbiota is a source of sleep-promoting signals. Bacterial metabolites and components of the bacterial cell wall are likely to provide important links between the intestinal commensal flora and sleep-generating mechanisms in the brain. Butyrate is a short-chain fatty acid produced by the intestinal bacteria by the fermentation of nondigestible polysaccharides. We tested the hypothesis that butyrate may serve as a bacterial-derived sleep-promoting signal. Oral gavage administration of tributyrin, a butyrate pro-drug, elicited an almost 50% increase in non-rapid-eye movement sleep (NREMS) in mice for 4 hours after the treatment. Similarly, intraportal injection of butyrate led to prompt and robust increases in NREMS in rats. In the first 6 hours after the butyrate injection, NREMS increased by 70%. Both the oral and intraportal administration of butyrate led to a significant drop in body temperature. Systemic subcutaneous or intraperitoneal injection of butyrate did not have any significant effect on sleep or body temperature. The results suggest that the sleep-inducing effects of butyrate are mediated by a sensory mechanism located in the liver and/or in the portal vein wall. Hepatoportal butyrate-sensitive mechanisms may play a role in sleep modulation by the intestinal microbiota.

## Introduction

Sleep is greatly affected by peripheral metabolic signals, such as satiety and orexigenic hormones^1–3^, increased lipolysis^4,5^, systemic pro-inflammatory signals^6^, activation of brown adipose tissue^7^ or the liver^8^. Recent evidence points to the importance of the intestinal microbiota in metabolic signaling (reviewed in^9^). Microbiota-derived signals modulate complex brain-related functions and various behaviors (reviewed in^10^).

The brain sleep mechanisms and the gut flora are linked through a dynamic bidirectional relationship. Depletion of intestinal microbiota induces significant reduction in sleep suggesting that the gut flora is a source of sleep-inducing signals^11,12^, while circadian disruption and chronic sleep fragmentation promote intestinal dysbiosis^13,14^. Cell wall components of bacteria induce sleep when injected systemically^15–18^ suggesting that fragments of disintegrating intestinal bacteria, once translocated into the portal circulation, could serve in sleep signaling. In addition to the cell wall fragments, live intestinal bacteria are also a source of biologically active metabolites, such as short-chain fatty acids (SCFAs), secondary bile acids, indole-derivatives, succinate or hormones and neurotransmitters (reviewed in^19^). The role of metabolites produced by live bacteria in sleep regulation is, however, poorly understood.

Butyrate, a SCFA, is a product of anaerobic bacterial fermentation of non-digestible carbohydrates in the hindgut, a major metabolic product of the clostridial clusters of intestinal flora^20^. Mammalian cells do not produce significant amounts of butyrate, the only significant sources are the microbiota and ingestion of dairy products^21,22^. Butyrate binds to the FFAR2, FFAR3 and GPR109A receptors^23–27^, and it also acts as a histone deacetylase inhibitor and affects gene transcription^28^. It is readily absorbed into the portal circulation and transported directly to the liver^29^. The liver represents a major sink for intestinally-produced butyrate as evidenced by the steep concentration gradient between portal and systemic levels of butyrate^29–31^. All three butyrate receptors are expressed in the liver^23,32,33^.

We hypothesized that intestinally-produced butyrate acts on hepatoportal sensory mechanisms to promote sleep. To test this, we investigated the effects of oral administration, direct intraportal injection, as well as systemic injection of butyrate and tributyrin, a butyrate-yielding pro-drug, in mice and rats. Our results demonstrate that oral and intraportal administration of butyrate induces robust increases in non-rapid-eye movement sleep (NREMS), while systemic butyrate treatment has no effect on sleep. These findings indicate the existence of a butyrate-sensitive hepatoportal sleep-inducing sensory mechanism.

## Results

### Oral gavage administration of tributyrin

**Figure 1:**
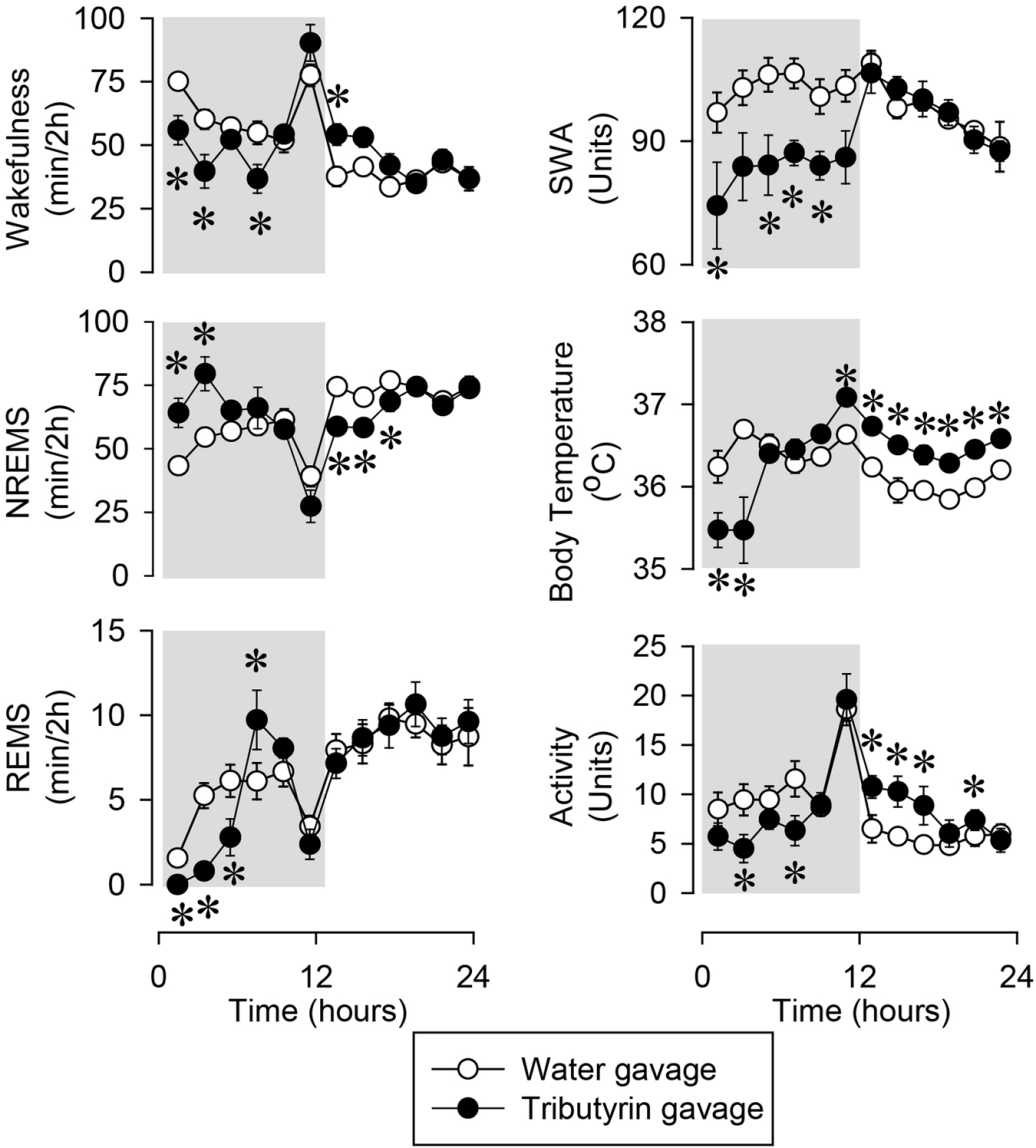
The effects of oral administration of tributyrin on wakefulness, non-rapid-eye movement sleep (NREMS), rapid-eye movement sleep (REMS), electroencephalographic (EEG) slow-wave activity (SWA) motor activity and body temperature. Data are presented in 2-h time blocks; shaded area represents the dark period. Time “0”: time of the treatments. Asterisks: significant difference from baseline, Tukey’s HSD test; error bar: SE.

Oral gavage administration of tributyrin at the beginning of the dark phase elicited robust sleep responses in mice (Fig. 1, Table 1). In the first four hours after the treatment, time spent in NREMS increased by 47% above baseline at the expense of rapid-eye movement sleep (REMS) and wakefulness (NREMS baseline: 97.9 ± 3.3 min/4 h, tributyrin: 143.7 ± 10.0 min/4 h, p < 0.01; REMS baseline: 6.8 ± 1.0 min/4 h, tributyrin: 0.8 ± 0.3 min/4 h, p < 0.001). The increases in NREMS time were due to significantly longer NREMS episodes, while REMS decreases were the consequence of a significant decrease in the number of REMS episodes [average episode numbers in the first 6 h on the baseline day: NREMS 34 ± 1.7, REMS 9 ± 1.1; after tributyrin treatment: NREMS 32 ± 3.2, REMS 2 ± 0.7 (vs. baseline p < 0.001); average episode durations in the first 6 h on the baseline day: NREMS 273 ± 14.2 s, REMS 89 ± 5.7 s; after tributyrin treatment: NREMS 407 ± 40.9 min (vs. baseline p < 0.001), REMS 66 ± 15.5 s]. Sleep latency was not affected (baseline: 18.3 ± 4.0 min, tributyrin: 21.3 ± 3.0 min).

**Table 1:** Oral administration of tributyrin.

	NREMS			REMS			Temperature			Activity			SWA		
	*df*	*F*	*p*	*df*	*F*	*p*	*df*	*F*	*p*	*df*	*F*	*p*	*df*	*F*	*p*
*Treatment*	1,7	0.7	n.s.	1,7	1.4	n.s.	1,7	2.4	n.s.	1,7	0.0	n.s.	1,7	14.6	<0.01
*Time*	11,77	15.12	<0.001	11,77	18.3	<0.001	11,77	7.5	<0.001	11,77	11.7	<0.001	11,77	2.7	<0.01
*Treatment x Time*	11,77	6.1	<0.001	11,77	4.0	<0.001	11,77	12.8	<0.001	11,77	3.2	<0.01	11,77	3.4	<0.001

Non-rapid eye movement sleep (NREMS), rapid-eye movement sleep (REMS), body temperature, motor activity and electroencephalographic slow-wave activity (SWA): statistical results.

The NREMS increase was accompanied by a 0.8–1.2 °C drop in body temperature and greatly suppressed electroencephalographic slow-wave activity (EEG SWA) and motor activity. Subsequently, there was a short rebound increase in REMS during the second half of the dark, and a decrease in NREMS in the first half of the light period. Body temperature and activity were slightly, but significantly, elevated in the light period.

### Intraportal administration of sodium butyrate

**Figure 2:**
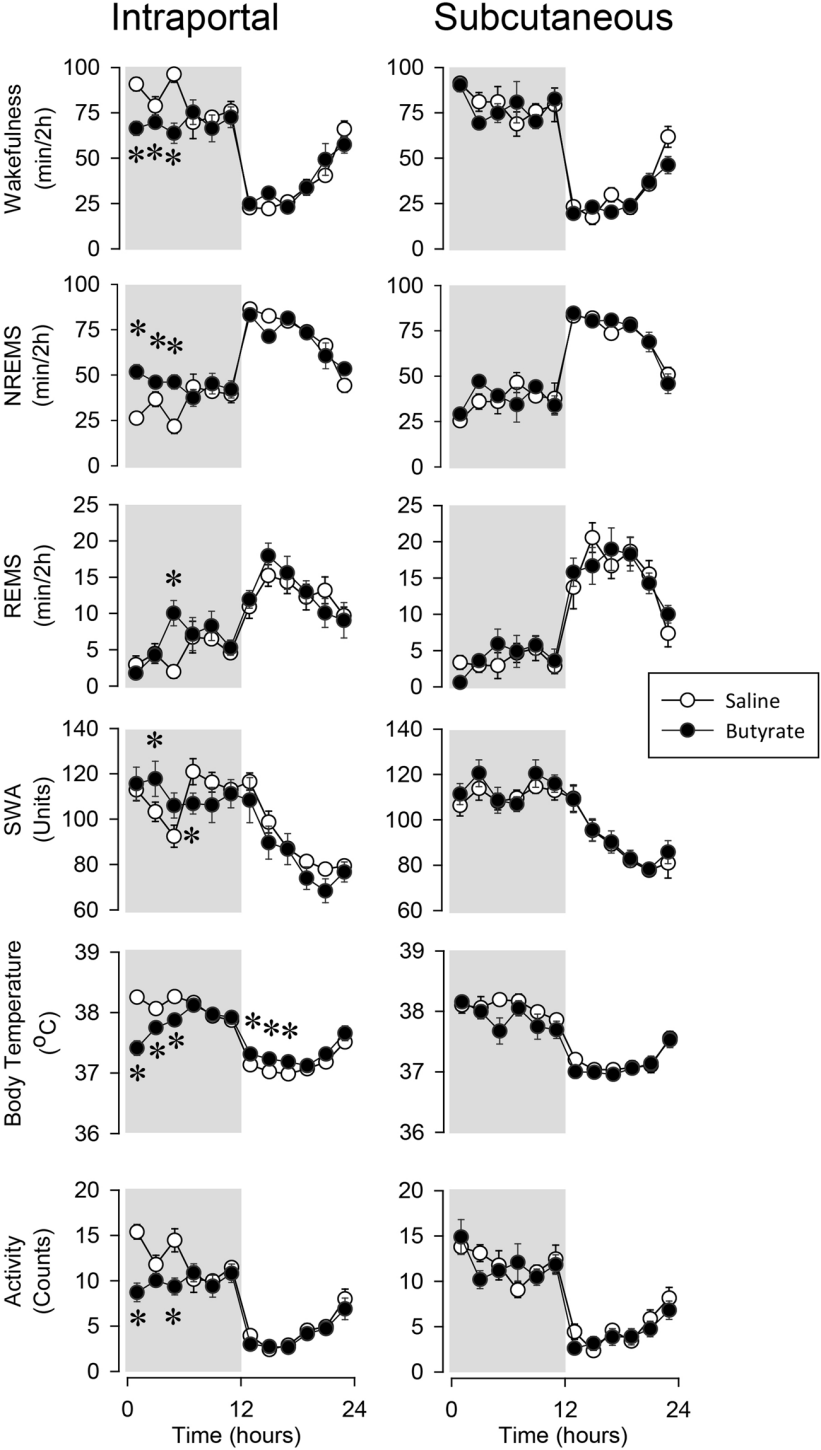
The effects of intraportal and subcutaneous administration of butyrate on wakefulness, NREMS, REMS, EEG SWA, motor activity and body temperature. See legend to Fig. 1 for details.

Intraportal injection of butyrate induced prompt and robust NREMS increases in rats (Fig. 2, Table 2). Sleep latency decreased from the baseline value of 36.1 ± 6.7 to 2.7 ± 2.0 min after butyrate treatment (p < 0.01). Overall, NREMS increased by 70% in the first 6 hours after the butyrate injection (baseline: 84.6 ± 6.7 min/6 h, butyrate: 144.0 ± 4.2 min/6 h). The animals showed the behavioral signs of normal sleep, they were easily arousable, and actively engaged with their environment in response to mild tactile or auditory stimuli. The NREMS response was due to the increased duration of NREMS episodes, while the number of sleep episodes was not affected (average NREMS episode duration in the first 6 h on the baseline day: 186 ± 16.2 s, after butyrate treatment: 301 ± 17.4 s, p < 0.001; number of NREMS episodes in the first 6 h on the baseline day: 28 ± 2.7, after butyrate treatment: 29 ± 1.9).

**Table 2:** Intra-portal administration of butyrate.

	NREMS			REMS			Temperature			Activity			SWA		
	*df*	*F*	*p*	*df*	*F*	*p*	*df*	*F*	*p*	*df*	*F*	*p*	*df*	*F*	*p*
*Treatment*	1,7	67.7	<0.001	1,7	7.8	<0.05	1,8	2.0	n.s.	1,7	0.2	n.s.	1,7	0.2	n.s.
*Time*	11,77	38.6	<0.001	11,77	10.2	<0.001	11,88	54.0	<0.001	11,77	37.2	<0.001	11,77	37.2	<0.001
*Treatment x Time*	11,77	4.3	<0.001	11,77	2.5	<0.01	11,88	14.0	<0.001	11,77	3.7	<0.001	11,77	3.7	<0.001

NREMS, REMS, body temperature, motor activity and EEG SWA: statistical results.

REMS also increased after butyrate treatment as indicated by significant treatment and treatment x time interactions in ANOVA; *post hoc* analysis revealed significant REMS increase in the 5–6 h time block. REMS increase was due to the combined effects of slightly elevated episode numbers and episode durations; neither change, by itself, was significant (average REMS episode duration in the first 6 h on the baseline day: 79 ± 9.7 s, after butyrate treatment: 95 ± 8.0 s; number of REMS episodes in the first 6 h on the baseline day: 6 ± 1.3, after butyrate treatment: 10 ± 1.4).

Motor activity decreased by 39% and body temperature by 0.4–1 °C in the first 6 h. The effects on EEG SWA were biphasic. In the first 6 h after the butyrate injection, there was a tendency towards increased SWA, which was followed by a slight, but significant decline below baseline.

### Systemic administration of sodium butyrate

**Figure 3:**
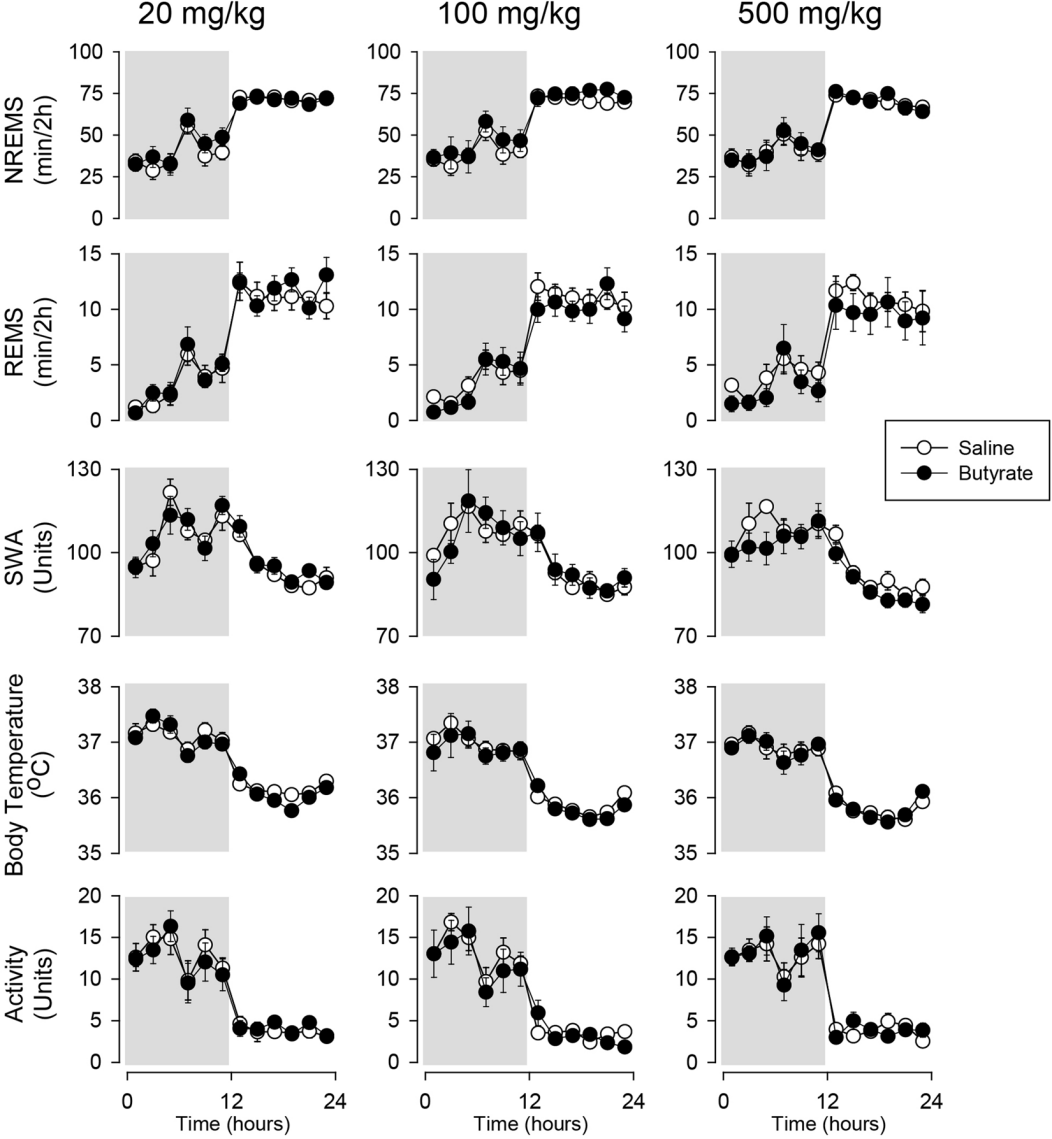
The effects of intraperitoneal administration of butyrate on NREMS, REMS, EEG SWA, motor activity and body temperature. See legend to Fig. 1 for details.

Systemic injection of butyrate did not have any significant effects on sleep, EEG SWA, body temperature and motor activity. In rats, subcutaneous injection of 1 g/kg, the same dose that promotes sleep after intraportal administration, did not affect any of the measured parameters (Fig. 2, Table 3). Similarly, in mice, intraperitoneal injection of 0.02, 0.1 and 0.5 g/kg butyrate was void of any significant effects (Fig. 3, Table 4).

**Table 3:** Subcutaneous administration of butyrate.

	NREMS			REMS			Temperature			Activity			SWA		
	*df*	*F*	*p*	*df*	*F*	*p*	*df*	*F*	*p*	*df*	*F*	*p*	*df*	*F*	*p*
*Treatment*	1,4	0.2	n.s.	1,4	2.1	n.s.	1,4	6.6	0.06	1,4	1.4	n.s.	1,4	0.4	n.s.
*Time*	11,44	42.9	<0.001	11,44	18.1	<0.001	11,44	53.1	<0.001	11,44	21.1	<0.001	11,44	16.6	<0.001
*Treatment x Time*	11,44	1.0	n.s.	11,44	1.2	n.s.	11,44	1.6	n.s.	11,44	1.4	n.s.	11,44	0.5	n.s.

NREMS, REMS, body temperature, motor activity and EEG SWA: statistical results.

**Table 4:** Intraperitoneal administration of butyrate.

	NREMS			REMS			Temperature			Activity			SWA		
	*df*	*F*	*p*	*df*	*F*	*p*	*df*	*F*	*p*	*df*	*F*	*p*	*df*	*F*	*p*
***20 mg/kg***															
*Treatment*	1,6	4.6	n.s.	1,6	1.8	n.s.	1,6	0.8	n.s.	1,5	0.9	n.s.	1,7	0.5	n.s.
*Time*	11,66	26.4	<0.001	11,66	47.1	<0.001	11,66	60.7	<0.001	11,55	17.1	<0.001	11,77	14.2	<0.001
*Treatment x Time*	11,66	0.8	n.s.	11,66	0.8	n.s.	11,66	1.8	n.s.	11,55	0.4	n.s.	11,77	0.8	n.s.
***100 mg/kg***															
*Treatment*	1,6	2.1	n.s.	1,6	1.8	n.s.	1,6	0.3	n.s.	1,6	0.6	n.s.	1,5	0.0	n.s.
*Time*	11,66	19.8	<0.001	11,66	26.4	<0.001	11,66	43.5	<0.001	11,66	33.6	<0.001	11,55	7.7	<0.001
*Treatment x Time*	11,66	0.4	n.s.	11,66	1.1	n.s.	11,66	0.5	n.s.	11,66	0.6	n.s.	11,55	0.8	n.s.
***500 mg/kg***															
*Treatment*	1,6	0.0	n.s.	1,6	1.5	n.s.	1,6	0.2	n.s.	1,6	0.0	n.s.	1,5	3.9	n.s.
*Time*	11,66	1.1	n.s.	11,66	6.5	<0.001	11,66	36.5	<0.001	11,66	18.1	<0.001	11,55	9.4	<0.001
*Treatment x Time*	11,66	0.6	n.s.	11,66	1.8	n.s.	11,66	1.3	n.s.	11,66	0.3	n.s.	11,55	0.8	n.s.

NREMS, REMS, body temperature, motor activity and EEG SWA: statistical results.

## Discussion

Evidence suggests that the gut bacteria are a source of sleep-inducing signals^11,12^, and we hypothesized that SCFAs may serve as such signal. Our major finding is that orally or intraportally administered tributyrin and butyrate, respectively, robustly increases NREMS in rats and mice. These observations are consistent with prior reports that intravenous injection of butyrate induces slow, high-amplitude EEG waves and behavioral signs of sleep in rabbits^34^ or EEG-defined NREMS in cats^35^.

Butyrate is a four-carbon SCFA, produced by the microbiota in mice and rats. It is the product of the anaerobic fermentation of non-digestible carbohydrates by gut bacteria and also a component of dairy products, such as butter, milk and cheese^36^. Tributyrin is an ester, composed of three butyric acid molecules and glycerol. It is considered a pro-drug to deliver biologically active butyrate as lipases in the host organism hydrolyze it resulting in the release of butyrate^37^. SCFAs, including butyrate, are readily absorbed from the intestines into the portal circulation and directly reach the liver^29^. Plasma levels of butyric acid in the portal circulation after oral administration of tributyrin are higher and more prolonged without detectable toxicity in mice and rats as compared to administration of butyrate itself^38^. To mimic the effects of intestinally produced butyrate, we administered tributyrin orally to mice. This treatment elicited an almost 50% increase in NREMS in the first 4 h supporting the notion that butyrate from the intestinal tract may potentially serve as a sleep-inducing signal molecule. NREMS increased at the expense of both REMS and wakefulness. REMS suppression may be due to the mutual inhibitory interaction between NREMS- and REMS-promoting mechanisms^39^ or it could be a NREMS-independent effect of tributyrin. The treatments were administered at dark onset. Since the latency to increased sleep is very short and the duration of the sleep increases did not exceed 8 hours, the effects of butyrate on sleep were manifested predominantly during the dark, active, phase.

Sleep-inducing doses of butyrate also elicited a 0.4–1.2 °C drop in body temperature. Since naturally occurring NREMS is associated by decreased energy expenditure and body temperature (reviewed in^40^), it is possible that the slight hypothermic response is simply the thermic manifestation of enhanced NREMS after butyrate treatment. It has been proposed that a drop in core body temperature prompts sleepiness^41^, thus an alternative interpretation is also possible, *i.e*., an initial drop in body temperature in response to butyrate may invoke the sleep responses.

Baseline sleep recordings were performed on the day before the butyrate treatment. In thoroughly-habituated rats and mice, such as our experimental animals, sleep and body temperature are remarkably stable across two successive days. This is evidenced, for example, by the experiments where mice received ip injection of saline on day 1 and butyrate on the following day (Fig. 3). Thus, it is highly unlikely that the observed sleep-promoting effects of intraportally or orally administer butyrate are confounded by order of the treatments.

There is a steep concentration gradient between the high portal levels of butyrate and very low butyrate concentration in the systemic circulation^29,30,42,43^, and orally administered tributyrin increases portal, but not systemic, levels of butyrate^44^. These observations indicate that the liver removes almost all butyrate from the portal blood^31^. Thus, the most likely target for butyrate to induce sleep is the hepatoportal system. To investigate this possibility, we injected butyrate directly into the portal vein in rats. Intra-portal butyrate treatment greatly reduced NREMS latency and increased the time spent in NREMS. To investigate, if a potential butyrate overflow from the liver into the systemic circulation could be responsible for the sleep effects, we injected the same amount of butyrate systemically. Systemic administration of butyrate did not have any effect on sleep in rats. Similarly, none of the systemically-administered doses of butyrate had any effect on sleep-wake activity in mice. These findings indicate that the sleep effects of orally or intraportally administered butyrate are not due to the actions of butyrate that possibly escaped the hepatic sink. We conclude that butyrate acts on the liver and/or the portal vein to promote NREMS. There is prior evidence that the liver is involved in peripheral sleep signaling, since local warming of the liver increases NREMS^8^, and depletion of liver Kupffer cells impairs recovery sleep responses after sleep loss and sleep in a cold environment^45^.

Hepatoportal sensors have been described for several gut-derived molecules, *e.g*., glucose, cholecystokinin, and amino acids^46–48^. They are located in the wall of the portal vein and the liver and have been implicated in the regulation of glucose and energy homeostasis (reviewed in^49^). There is also evidence for hepatoportal SCFA sensors. Butyrate receptors are present in the hepatoportal region. Butyrate signals through the receptors FFAR2, FFAR3 and GPR109A, all of which is expressed in the liver^32,33,50^, thus may serve as hepatic sensors. FFAR3 is also expressed by the portal vein wall in close proximity to neuronal markers^51^. The activation of these receptors affects brain circuits as evidenced by the observation that the effects of dietary SCFAs on the activity of the nucleus tractus solitarius and parabrachial nucleus are abolished by the selective sensory denervation of the periportal area^51^. The sensory innervation of the hepatoportal region is provided by the vagus and spinal afferents, both of which have been implicated in sleep signaling^52–55^. Butyrate directly activates vagal afferents^56^ and the effects of butyrate on feeding is suppressed by hepatic vagotomy^57^.

Several bacterial-derived sleep-inducing molecules, such as lipopolysaccharide and fragments of peptidoglycans, have been described before (reviewed in^58^). All these molecules are components of the bacterial cell wall, they are released from disintegrating bacteria or from bacteria during cell division. They have pronounced inflammatory actions via the stimulation of the production of pro-inflammatory cytokines (reviewed in^59^). Sleep responses to systemic bacterial infection are linked to these pro-inflammatory processes. The properties of butyrate are, however, fundamentally different. Butyrate is produced by live bacteria in the intestines, and it has strong anti-inflammatory properties. It suppresses colonic and liver inflammation and lipopolysaccharide-induced production of pro-inflammatory cytokines and NF-κB activation^26,60–63^. This indicates that not only systemic pro-inflammatory signals related to bacterial infections, but also bacterial-derived anti-inflammatory signals from the intestinal tract have the potential to modulate sleep.

## Methods

### Animals

Male Sprague-Dawley rats and breeding pairs of C57BL/6 J mice were purchased from The Jackson Laboratories, Inc.; the mice were further bred at Washington State University. During the experiments, the animals were housed in temperature-controlled (mice: 30 ± 1 °C, rats: 23 ± 1 °C), sound-attenuated isolation chambers on a 12:12-hour light-dark cycle (lights on at 3 AM). Food and water were available *ad libitum* throughout all experiments. Animals were provided regular lab chow (Harlan Teklad, Product no. 2016), in which fats, proteins, and carbohydrates comprise 12%, 22%, and 66% of calories, respectively. All animal procedures were conducted in compliance with the recommendations in the Guide for the Care and Use of Laboratory Animals of the National Institutes of Health. All animal protocols were approved by the Institutional Animal Care and Use Committees at Washington State University.

### Surgery

All surgical procedures were performed using ketamine-xylazine anesthesia (87 and 13 mg/kg, respectively). For sleep-wake activity recordings, 3-month old mice (25.5 ± 0.9 g) and rats (325–350 g) were implanted with three cortical EEG electrodes, placed over the frontal and parietal cortices, and two nuchal electromyographic (EMG) electrodes. The EEG and EMG electrodes were anchored to the skull with dental cement. Telemetry transmitters were implanted intraperitoneally for body temperature and motor activity recordings. In addition, the rats were implanted with an intraportal cannula^64^ three weeks prior to the sleep surgery. Briefly, a biocompatible polyurethane was inserted into the superior mesenteric vein and the tip of the cannula routed to the main stream of the portal vein. The free end of the cannula was routed subcutaneously to the dorsal surface of the neck and exteriorized. The cannula was sutured to the portal vein, the abdominal muscles and the neck skin. The patency was maintained by daily flushing with 0.2 ml isotonic saline followed by 0.08 ml of lock solution containing 500 IU/ml heparin in 50% glycerol solution. The animals were allowed to recover from surgery for at least 10 days before any experimental manipulation started and handled daily to adapt them to the experimental procedures.

### Sleep-wake activity recordings and analyses

The animals were tethered to commutators, which were further routed to Grass Model 15 Neurodata amplifier system (Grass Instrument Division of Astro-Med, Inc., West Warwick, RI). The amplified EEG and EMG signals were digitized at 256 Hz and recorded by computer. The high-pass and low-pass filters for EEG signals were 0.5 and 30.0 Hz, respectively. The EMG signals were filtered with low and high cut-off frequencies at 100 and 10,000 Hz, respectively. The outputs from the 12A5 amplifiers were fed into an analog-to-digital converter and collected by computer using Sleep Wave software (Biosoft Studio, Hersey, PA). Sleep-wake states were scored visually off-line in 10-s segments. The vigilance states were defined as NREMS, REMS and wakefulness according to standard criteria as described previously^1^. EEG power data from each artifact free 10-s segment were subjected to off-line spectral analysis by fast Fourier transformation. EEG power data in the range of 0.5 to 4.0 Hz during NREMS were used to compute EEG SWA. EEG SWA data were normalized for each animal by using the average EEG SWA across 24 h on the baseline day as 100.

### Telemetry recordings

Core body temperature and locomotor activity were recorded by MiniMitter telemetry system (Starr Life Sciences Corp.) using VitalView software. Temperature and activity values were collected every 1 and 10 min, respectively, throughout the experiment and were averaged over 2-h time blocks.

### Experimental procedures

#### Experiment 1: The effects of oral gavage administration of tributyrin in mice

Eight mice were habituated to the gavage procedure by administering 0.3 ml water for 7 days 5–15 min before dark onset. After the habituation period, a baseline day was recorded after the oral gavage of 0.3 ml water. This treatment controls for the non-specific effects of the gavage administration, such as changes in the gastric volume. The following day, 0.3 ml tributyrin was administered (Millipore Sigma). The treatments were performed 5–10 min before dark onset. Sleep and telemetric recordings started at dark onset and continued for 23.5 h.

#### Experiment 2: The effects of intraportal administration of sodium butyrate in rats

Ten rats were habituated to the injection procedure by daily flushing of the cannula with isotonic saline 5–20 min before dark onset. On the baseline day, 2 ml/kg isotonic NaCl (vehicle) was administered through the cannula. On the test day, the animals received 1 g/kg sodium butyrate, dissolved in isotonic NaCl, in a volume of 2 ml/kg. The pH of the butyrate solution was set to 7.4 by using NaOH. The treatments were performed 5–20 min before dark onset. Sleep and telemetric recordings started at dark onset and continued for 23.5 h. Due to the malfunction of some of the implants, EEG/EMG was obtained only from 8 animals, and body temperature from 9 rats.

#### Experiment 3: The effects of subcutaneous administration of sodium butyrate in rats

Ten days after Experiment 2, five rats were used again to test the effects of sc administration of 1 g/kg butyrate. The animals were habituated to the treatment by daily sc administration of isotonic saline. On the baseline day, the animals were injected sc with 2 ml/kg isotonic NaCl (vehicle). On the test day, the animals received 1 g/kg buffered sodium butyrate subcutaneously in a volume of 2 ml/kg. The treatments took place 5–10 min before dark onset. Sleep and telemetric recordings started at dark onset and continued for 23.5 h.

#### Experiment 4: The effects of intraperitoneal administration of sodium butyrate in mice

Three doses of butyrate were tested in the same group of mice (n = 7). After the habituation period, the animals received 10 ml/kg isotonic NaCl (vehicle) ip to obtain baseline values. On the test day, the mice were injected with 20 mg/kg sodium butyrate intraperitoneally. One week later, a new vehicle baseline day was recorded followed by the test day of 100 mg/kg butyrate. Finally, after one additional week of recovery, a third vehicle baseline and test day (500 mg/kg butyrate) were recorded. The treatments took place 5–10 min before dark onset. Sleep and telemetric recordings started at dark onset and continued for 23.5 h.

### Statistics

Time spent in wakefulness, NREMS and REMS, as well as, EEG SWA, body temperature and motor activity were calculated in 2-h blocks. Two-way repeated measures ANOVA was performed across 24 h between test days and the corresponding baselines (factors: treatment and time, both repeated). When appropriate, Tukey’s HSD test was applied *post hoc*. An α-level of P < 0.05 was considered to be significant.

## Data Availability

All data generated or analyzed during this study are included in this published article.
